# Multiple drug resistance in the canine hookworm *Ancylostoma caninum*: an emerging threat?

**DOI:** 10.1186/s13071-019-3828-6

**Published:** 2019-12-09

**Authors:** Pablo D. Jimenez Castro, Sue B. Howell, John J. Schaefer, Russell W. Avramenko, John S. Gilleard, Ray M. Kaplan

**Affiliations:** 1 Department of Infectious Diseases, College of Veterinary Medicine, University of Georgia, Athens, GA 30602 USA; 2 Grupo de Parasitología Veterinaria, Universidad Nacional de Colombia, Bogotá, Colombia; 3 School of Veterinary Medicine, Department of Biomedical and Diagnostic Sciences, University of Tennessee, Knoxville, USA; 4 Department of Comparative Biology and Experimental Medicine, Faculty of Veterinary Medicine, University of Calgary, Calgary, Alberta Canada

**Keywords:** *Ancylostoma caninum*, Hookworms, Resistance, Anthelmintics, Canine health

## Abstract

**Background:**

The canine hookworm, *Ancylostoma caninum* is the most prevalent and important intestinal nematode parasite of dogs in the USA. Hookworms are typically well controlled by treatment with all commonly used anthelmintics that are approved for this use in dogs. However, in the past few years, cases of recurrent/persistent canine hookworm infections appear to have dramatically increased, suggesting that anthelmintic resistance (AR) may have evolved in this parasite. These cases are highly overrepresented by greyhounds, but multiple other breeds are also represented. The aim of this study was to characterize several of these suspected resistant isolates using *in vitro*, genetic and clinical testing to determine if these cases represent true anthelmintic resistance in *A. caninum*.

**Methods:**

Fecal samples containing hookworm eggs from three cases of persistent hookworm infections; one from a greyhound, one from a miniature schnauzer and one from a hound-mix, were received by our laboratory. These were then used to establish infections in laboratory dogs and to perform egg hatch assays (EHA) and larval development assays (LDA) for detecting resistance to benzimidazoles and macrocyclic lactones, respectively. Additional EHA and LDA were performed on eggs recovered from the laboratory-induced infections. Fecal egg count reduction tests were performed to detect resistance to pyrantel. Deep amplicon sequencing assays were developed to measure the frequency of non-synonymous single nucleotide polymorphisms (SNP) at codons 167, 198 and 200 of the *A. caninum* isotype-1 β-tubulin gene.

**Results:**

Resistance ratios for the three *A. caninum* isolates tested ranged from 6.0 to > 100 and 5.5 to 69.8 for the EHA and LDA, respectively. Following treatment with pyrantel, reduction in faecal egg counts was negative or 0%. Deep amplicon sequencing of the isotype-1 β-tubulin gene identified a high frequency of resistance-associated SNPs at codon 167 in all three resistant isolates and in two additional clinical cases.

**Conclusions:**

These data conclusively demonstrate multiple anthelmintic resistance in multiple independent isolates of *A. caninum*, strongly suggesting that this is an emerging problem in the USA. Furthermore, evidence suggest that these resistant hookworms originate from racing greyhound farms and kennels, though additional research is needed to confirm this. 
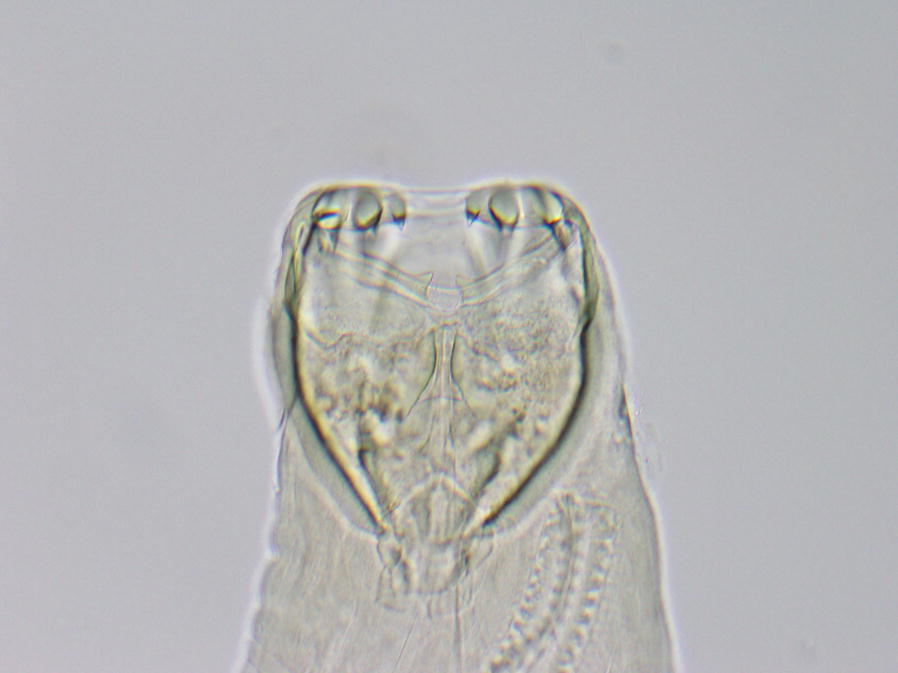

## Background

The canine hookworm, *Ancylostoma caninum*, is the most prevalent and important intestinal nematode parasite of dogs in the USA [1]. A recent study evaluating over 39 million fecal samples from 2012–2018, showed evidence of a steady yearly increase in prevalence from 2015–2018, with an overall increase of 47% [2]. Anthelmintic drugs approved for the treatment of *A. caninum* in the USA include, febantel, moxidectin, milbemycin oxime, fenbendazole and pyrantel. In registration studies, febantel, moxidectin and milbemycin oxime all demonstrated efficacy of > 99% [3–5], fenbendazole demonstrated efficacy of > 98% [6] and pyrantel demonstrated a somewhat variable efficacy, with a mean across studies of approximately 94%, where more than half of those studies yielded > 99% [7]. Pathological consequences of hookworm infection include iron-deficiency anemia, hypoalbuminemia and enteritis, characterized by diarrhea that may contain fresh (hematochezia) or digested blood (melena) [8–10].

Hookworms are very successful parasites and one of the main reasons is the multiple routes by which they can infect their hosts. *Ancylostoma caninum* is transmitted by the transmammary route to newborn puppies [11], percutaneously [12], orally [9], or *via* ingestion of paratenic hosts, such as rodents [13] and insects [14]. Transmammary infection results from reactivation of arrested tissue-stage larvae in pregnant bitches, which then travel to the mammary glands, where they are passed in the colostrum and milk to newborn puppies for up to 18 days [15].

In puppies infected *via* skin penetration there is a blood-lung migration pathway [16, 17]. However, in older dogs, this pathway and developmental cycle is substantially modified; rather than the lungs, most of the larvae penetrate peripheral organs (somatic tissues) such as muscle (M.D. Little, unpublished observations) or gut wall [18], where they enter into an arrested state and are capable of surviving for several years [19].

An interesting biological feature of *A. caninum* infection is the phenomenon known as “larval leak”, where arrested somatic larvae continuously migrate to the small intestine where they develop to the adult stage [9, 19]. These cases are not associated with pregnancy and dogs with “larval leak” will chronically shed hookworm eggs, often in low numbers, with treatment only providing a temporary break of egg shedding, due to new reactivated hypobiotic larvae repopulating the gut and beginning a new round of egg shedding within a few weeks of treatment [16]. The actual mechanism responsible for this phenomenon is thought to be an immunological deficit; however, a specific cause has not been elucidated [20].

Because this larval reactivation is a well-described phenomenon, dogs presenting with recurrent hookworm infections are presumed to be suffering from this problem. Even though, no data are available to document the historical number of cases of recurrent hookworm infection in dogs, parasitologists at several veterinary colleges in the USA who we have communicated with, including our laboratory, have received increasing numbers of communications in the last 2–3 years. These cases are heavily overrepresented by greyhounds, but include many other breeds as well. The emergence of anthelmintic resistance in *A. caninum* would give a plausible explanation for these recent observations.

Parasitic strongylid nematodes have a number of genetic features, which favour the development of anthelmintic resistance, such as rapid rates of nucleotide sequence evolution and exceedingly large effective population sizes, leading to remarkably high levels of genetic diversity [21, 22]. Anthelmintic resistance is a heritable trait [23], and is defined as occurring ‘when a greater frequency of individuals in a parasite population, usually affected by a dose or concentration of compound, are no longer affected, or a greater concentration of drug is required to reach a certain level of efficacy [24]. *Ancylostoma caninum* is the most common nematode parasite of greyhounds on breeding farms [25]; this high prevalence is likely a consequence of the unrestricted access to exercise runs made out of sand and dirt, which produces an ideal environment for the development and survival of the infective larvae [16]. To address the problem of nematode infections, the dogs on these breeding farms are subject to a very intense deworming protocol; puppies are often treated weekly with an anthelmintic until three months of age, then tri-weekly until sixth months of age, and then monthly for the rest of their breeding or racing lives [25]. This would present a very high drug selection pressure on the hookworm population on these farms and racing kennels.

In livestock, the intensive use and near complete reliance on anthelmintic drugs for control of nematode infections has led to high levels of anthelmintic resistance and multi-drug resistant (MDR) populations of nematodes on a global scale [26]. In contrast, anthelmintic resistance in *A. caninum* has developed much more slowly, with few cases reported, and until this year, only to pyrantel. The first report of pyrantel resistance was from New Zealand in a greyhound puppy that was imported from Australia [27], with several more cases subsequently diagnosed in Australia [28–32]. The issue of whether resistance is likely to become a problem in parasites of dogs has received relatively little attention, and when addressed, it has been viewed as an issue relating to the increased use of prophylactic helminth treatments in pets [33]. However, the epidemiology of nematode transmission on greyhound farms much more closely resembles the epidemiological conditions present on livestock farms, than to the epidemiological conditions present in a pet home environment. Consequently, it would not be surprising if anthelmintic resistance also were to become a common problem on greyhound farms. Interestingly, coincident with our investigations, a recent publication reported resistance to benzimidazoles and macrocyclic lactones in an isolate of *A. caninum* obtained from a greyhound dog [34]. The dog in that case presented to a veterinary clinic with a hookworm infection that was subsequently refractory to multiple treatments with fenbendazole.

Beyond the concerns for canine health, multiple-drug resistance in canine hookworms could present serious public health concerns, since *A. caninum* is zoonotic. Humans infected percutaneously may develop *cutaneous larva migrans* (CLM) [35]. Cases of eosinophilic enteritis [36], as well as patent infections have also been described [37].

Given the increasing frequency of reports by veterinarians that our laboratory has been receiving of recurrent hookworm infections that are poorly responsive to anthelmintics, it seemed likely that anthelmintic resistance had evolved in *A. caninum*. The aim of this study was to characterize several of these suspected resistant isolates using *in vitro*, genetic, and clinical testing.

## Methods

### Parasite isolates

Three fecal samples containing hookworm eggs were received from veterinarians who were treating cases of recurrent hookworm infections in canine patients. These three “suspected-resistant” isolates of *A. caninum* were designated Worthy, Lacy and Tara. Two additional fecal samples from *A. caninum* isolates from dogs with no history of anthelmintic treatments were also received. One designated ETCR, was previously cycled in the laboratory and confirmed as susceptible, and a second was acquired from a local dog shelter, which was confirmed as susceptible during the study. For the experimental infections, eggs recovered from fecal samples were placed onto NGM plates [38] and cultured for seven days to obtain third-stage infective larvae, which were used subsequently to orally infect purpose-bred research dogs (University of Georgia AUP # A2017 10-016-Y1-A0).

In order to distinguish different passages and treatment events of the hookworm isolates, we established a naming convention as follows: name of the isolate followed by a number that corresponds to the number of passages the isolate has undergone. The letters F, P and M after the dot correspond to any treatments applied with either fenbendazole, pyrantel or milbemycin oxime, respectively. The number preceding the letter indicates the passage in which this treatment took place. For example, Worthy 4.1F2P3M would correspond to the fourth passage of the Worthy isolate and treatment with fenbendazole in the first passage, treatment with pyrantel in the second passage and treatment with milbemycin oxime in the third passage. Available diagnostic and treatment histories of the dogs from which we obtained the hookworm isolates are as follows.

#### Worthy

Three-year-old greyhound, adopted December 10, 2016 from Florida and currently residing in Tennessee. Prior to adoption, the dog was treated with pyrantel and administered heartworm prophylaxis (not specified).

- January 11, 2017: New pet exam at University of Tennessee College of Veterinary Medicine Community Practice Clinic, fecal-positive for hookworms. Administered fenbendazole (50 mg/kg) daily for 10 days and started monthly Heartgard® Plus (Merck, Kenilworth, NJ, USA) (ivermectin/pyrantel).
- January 31, 2017: Fecal-positive for hookworms. Administered fenbendazole (50 mg/kg) daily for 10 days.
- February 21, 2017: Fecal-negative.
- April 20, 2017: Fecal-positive for hookworms, reporting many eggs seen. Administered fenbendazole (50 mg/kg) daily for 10 days.
- July 26, 2017: Administered fenbendazole (50 mg/kg) daily for 10 days and switched from Heartgard® Plus (Merck) (ivermectin/pyrantel) to monthly Advantage Multi® (Bayer, Leverkusen, Germany) (imidacloprid/moxidectin).
- August 7, 2017: Administered fenbendazole (50 mg/kg) daily for 10 days.
- August 21, 2017: Fecal-positive for hookworms. Administered Advantage Multi® (Bayer, Leverkusen, Germany) (imidacloprid/moxidectin).
- September 21, 2017: Fecal-positive for hookworms. Administered Advantage Multi® (Bayer, Leverkusen, Germany) (imidacloprid/moxidectin).
- October 16, 2017: Fecal-positive for hookworms. Sample sent to the University of Georgia. Fecal egg count (FEC) of 160 eggs per gram (EPG).
- December 20, 2017: Research purpose-bred beagle was infected with 201 third-stage larvae.

#### Tara

Adult miniature schnauzer breeding bitch from St. Augustine, Florida.

- Spring 2017: Fecal examination was positive for hookworm eggs. Adult dogs started on Drontal® Plus (Bayer, Leverkusen, Germany) (praziquantel/pyrantel pamoate/febantel) once per month, with puppies receiving treatment at 2, 4, 6 and 8 weeks of age and then once per month afterwards. In addition, all dogs at this breeding kennel received Heartgard® Plus (Merck) (ivermectin/pyrantel) monthly. Therefore, all dogs were being treated twice monthly with pyrantel and once monthly with febantel.
- November 2017: Fecal examination positive for hookworms and sample sent to UGA. FEC of 100 EPG.
- December 20, 2017: Research purpose-bred beagle was infected with 250 third-stage larvae.

#### Lacy

Adult hound mix from Griffin, Georgia.

- Mid October-Mid November 2017: Treated twice, three weeks apart with a compounded combination of pyrantel, praziquantel and mebendazole.
- December 11, 2017: Dog was treated with a compounded combination of praziquantel, pyrantel, and oxantel.
- December 13 and December 15, 2017: Treated with pyrantel.
- December 16, 2017: Adult hookworm specimens were found whilst taking rectal temperature and hookworm eggs were present in feces. Treated with fenbendazole for 3 days (December 16–18, 2017).
- December 18, 2017: Fecal sample submitted to UGA containing live adult worms and eggs present in the feces. No FEC was performed.
- January 25, 2018: Research purpose-bred beagle was infected with 250 third-stage larvae.

#### ETCR

Susceptible laboratory isolate: from a naturally infected adult dog residing in Cumberland County, Tennessee, in June 2016 with a history of no anthelmintic treatments ever being given. This isolate had subsequent passages in research purpose-bred beagles and a sample was received at UGA on October 17, 2017, with further propagation in a research purpose-bred beagle.

#### Barrow

Susceptible laboratory isolate: a pooled fecal sample from an unknown number of naturally-infected adult shelter dogs residing in Barrow County, Georgia with no history of anthelmintic treatments. Sample was received at UGA on March 13, 2018. Research purpose-bred beagle was infected with 250 third-stage larvae on April 17, 2018.

#### In vitro assays

Fresh feces from laboratory beagles infected with the Worthy, Tara, Lacy, ETCR and Barrow isolates were collected and made into a slurry with water, followed by filtration through 425 µm and 180 µm sieves and then again through 85 µm and 30 µm nylon filters. The fecal material containing the eggs was then rinsed from the 30 µm filter with distilled water and reduced to a volume of 10–15 ml. This was then layered on top of saturated sucrose and centrifuged at 1372×*g* for 7 min at 4 °C. Following centrifugation, eggs were recovered, rinsed with distilled water through a 20 µm sieve, transferred to a tube and then the volume was adjusted to yield 50–60 eggs per 20 μl using distilled water.

#### Egg hatch assay (EHA)

Fresh feces containing undeveloped eggs were used, as partial egg development may affect the dose response [39]. Assays were performed using both agar and liquid-based methods with no significant difference detected between methods. Agar-based assays were performed using 96-well plates using a previously described agar-matrix technique [40] with minor modification. Liquid-based assays were also performed using a 96-well plate format [41] with minor modifications. A stock solution of 80 mM of thiabendazole (Sigma-Aldrich, St. Louis, MO, USA) was prepared using 100% dimethyl sulfoxide (DMSO, Sigma-Aldrich, St. Louis, MO, USA) and then was serially diluted using distilled water to produce 10 final concentrations ranging from 36 to 0.001125 μM in 1% DMSO. The first two wells of each row were negative controls containing only 0.5% DMSO for the agar plates and 1% DMSO for the liquid-based plates and wells 3–12 contained increasing concentrations of thiabendazole. Agar-based assay plates were prepared by adding 70 μl of 2% Agar (Bacto Agar, VWR, Becton Dickinson Sparks, MD, USA) and 70 μl of thiabendazole solution to each well. Liquid-based plates were prepared by just adding 100 μl of thiabendazole solution to each well with no agar. Agar plates were sealed with Parafilm (Bemis NA, Neenah, WI, USA) and stored in the refrigerator at 4 °C for a maximum of one week. Prior to performing the assays, plates were removed from the refrigerator and permitted to reach room temperature. Approximately 50–60 eggs in a volume of 10 μl were then added to each well. Plates were incubated for 48 h at 25 °C and assays were terminated by adding 20 μl of 10% Lugols iodine to all wells. Numbers of eggs and larvae in each well were counted and hatching was corrected for the average hatching rate in the control wells. The initial assays using ETCR, ETCR 1.0, Barrow, Tara, Lacy, Worthy, Worthy 1.1F and Worthy 2.1F were performed singly with each thiabendazole concentration tested in triplicate. In order to improve the precision of our measurement of IC_50_ and reduce the width of the 95% confidence intervals, we repeated the assays using three biological replicates of Barrow 1.0 and Worthy 4.1F3P, with three technical replicates per concentration in each assay.

#### Larval development assay (LDA)

Larval development assays were performed initially using DrenchRite® LDA (Microbial Screening Technologies, Armidale, New South Wales, Australia) assay plates [42]. The DrenchRite® LDA evaluates resistance to benzimidazoles, macrocyclic lactones and levamisole using the drugs, thiabendazole, ivermectin aglycone and levamisole, respectively. Subsequently, LDA plates were prepared using only ivermectin aglycone. The three-drug plates had concentrations of ivermectin aglycone ranging between 0.97–10,000 nM and the ivermectin aglycone-only plates had concentrations ranging between 1.9–1000 nM. After isolating the eggs as described for the EHA, 90 µl/ml of amphotericin B (250 μg/ml, supplied by Microbial Screening Technologies) were added and 20 μl containing approximately 50–70 eggs were dispensed into each well. Assay plates were sealed with Parafilm and incubated at 25 °C. After 24 h, 20 μl of nutritive media, composed of 0.87% Earle’s balanced salts, (Sigma-Aldrich, St. Louis, MO, USA), 1% yeast extract (BD Difco, VWR, Becton Dickinson Sparks, MD, USA), 0.76% NaCl (Sigma-Aldrich, St. Louis, MO, USA), with an addition of 1% *E. coli* OP50, were added to each well. The plates were resealed and incubated for 6 additional days, after which the assays were terminated by adding 20 μl of 50% Lugols iodine to all wells. The contents of each well were transferred to a clean 96-flat well plate and all eggs and larvae in each well were counted using an inverted microscope as previously described [43]. Development to L3 was corrected for all drug wells based on the average development in the control wells. The LDA does not evaluate pyrantel, which is the other anthelmintic approved for the treatment of hookworms of dogs in the USA. However, levamisole, which is used in the DrenchRite® plate, has a similar mechanism of action to pyrantel [44]. The initials assays performed with ETCR 1.0, Lacy and Worthy 1.0 were performed singly with each ivermectin concentration tested in duplicate. In order to improve the precision of our measurement of IC_50_ and reduce the width of the 95% confidence intervals, we repeated the assays using three biological replicates of laboratory isolates Barrow 1.0 and Worthy 4.1F3P, with two technical replicates per concentration in each assay.

### In vivo measurements

One laboratory dog infected with the initial Tara isolate (first passage) and two dogs infected with larvae from the second passage of the Worthy isolate (Worthy 2.1F), were treated orally with pyrantel (10 mg/kg, Strongid®, Parsippany-Troy Hills, NJ, USA). Reductions in fecal egg counts (FEC) were measured at day 10 for the Tara isolate and at day 13 for the Worthy isolate. The average of the FEC from the two dogs infected with the Worthy isolate was used for the reduction calculation. All FEC were performed in triplicate using the Mini-FLOTAC (University of Naples Federico II, Naples, Italy) procedure with a detection threshold of 5 EPG [45, 46], adding 2 g of feces to 18 ml of sodium nitrate (Feca-Med®, Vedco, Inc. St. Joseph; MO, USA, specific gravity = 1.2). Fecal egg count reduction was calculated using the following formula: (Pre-treatment FEC–Post-treatment FEC)/(Pre-treatment FEC) × 100. For the pre-treatment FEC, we used the 2-day mean of the day prior to treatment and the day of treatment or the average of the two days before treatment if FEC were not performed on the day of treatment.

### *Ancylostoma caninum* isotype-1 β-tubulin deep amplicon sequencing

DNA was extracted from pools of eggs, third-stage larvae or adults using a previously described lysis protocol [47]. Deep amplicon sequencing assays were developed to determine the frequency of non-synonymous single nucleotide polymorphisms (SNP) at codons 167, 198 and 200 of the *A. caninum* isotype-1 β-tubulin gene. The approach and methods were as previously described for ruminant trichostrongylid nematodes except for the primer design [48]. The presence of a large intron between exons 4 and 5 (1217 bp in reference sequence (GenBank: DQ459314.1) meant that a single amplicon encompassing the three codons of interest would be too long for reliable Illumina sequencing. Consequently, primers were designed to amplify two separate regions of the *A. caninum* isotype-1 β-tubulin gene; a 293 bp fragment between exons 3 and 4 encompassing codon 167 and a 340 bp fragment between exons 5 and 6 encompassing codons 198 and 200 (Table 1).

**Table 1:** *Ancylostoma* spp. isotype-1 β-tubulin primers

Primer	Sequence (5′–3′)	Length (bp)	Forward/reverse	Codons
ACB1_167_F	GGYGCAGGAAACAACTG	17	Forward	167
ACB1_167_R	CTTTGGTGAGGGGACAACA	19	Reverse	167
ACB1_200_F	GTRGTGGAGCCATACAATGC	20	Forward	198, 200
ACB1_200_R	GGCATGAAGAAGTGAAGACGT	21	Reverse	198, 200

Using these primers, adapted primers suitable for Illumina next-generation sequencing were designed as previously described [48]. The following PCR conditions were used to generate both fragments appropriate for sequencing: 5 μl of 5× NEB Q5 Reaction Buffer (New England Biolabs Ltd, Ipswich, MA, USA), 0.5 μl of 10 mM dNTPs, 1.25 μl of 10 μM Forward primer mixture, 1.25 μl of 10 μM Reverse primer mixture, 0.25 μl of NEB Q5 polymerase, 13.75 μl of molecular grade water and 3 μl of DNA lysate. The thermocycling parameters were 98 °C for 30 s, followed by 45 cycles of 98 °C for 10 s, 65 °C for 15 s, and 72 °C for 25 s, followed by 72 °C for 2 min. Samples were purified and barcoded primers added following the protocols outlined in Avramenko et al. [48]. Library preparation was as previously described and library sequencing performed using the Illumina MiSeq platform with the 2 × 250 v2 Reagent Kit (Illumina Inc., San Diego, CA, USA) [47]. The average read depth was ~14,000 for each sample fragment, ranging between 10,000 and 19,000 reads. Sequence analysis was performed following the bioinformatic pipeline outlined in Avramenko et al. [48]. Generated sequences were compared against a susceptible genotype *A. caninum* isotype-1 β-tubulin reference sequence (GenBank: DQ459314.1). Only observed variants resulting in non-synonymous changes at codons 167, 198 and 200 that are known to be associated with benzimidazole resistance in other strongylid nematodes are reported. The isolates examined were ETCR, Barrow, Worthy, Worthy 1.1F, Worthy 2.1F, Tara, Tara 1.1F and Lacy. Additionally, two clinical samples with a history of recurrent infections despite repeated anthelmintic treatments were included; Fame Taker (retired racing greyhound residing in Georgia) and Dolores (laboratory mix, Worthy’s housemate companion).

### *Ancylostoma caninum* ITS-2 rDNA deep amplicon sequencing

In order to confirm the hookworm species represented in the various samples, we used an ITS-2 rDNA deep amplicon sequencing assay [47]. This method is capable of discriminating between different nematode species based upon the sequence identity of the ITS-2 region of the rDNA. The samples were prepared and sequenced as described in Avramenko et al. [47] and analysed with the bioinformatic pipeline described in Avramenko et al. [49]. Several *A. caninum* and *A. braziliense* ITS-2 sequences were added to the analysis database for the purposes of this analysis (GenBank: DQ438050-DQ438054, DQ438060-DQ438062, DQ438065-DQ438067, AB751614-AB751616, DQ438072-DQ438079).

### Data analyses

All dose-response analyses were performed after log-transformation of the drug concentrations and constraining the bottom value to zero. Data were then fitted to a four-parameter non-linear regression algorithm with variable slope (GraphPad Prism® version 8.0, GraphPad Software, San Diego, CA, USA). The IC_50_ values, which represent the concentration of drug required to inhibit hatching (EHA) or development to the third larval stage (LDA) by 50% of the maximal response and corresponding resistance ratios (IC_50_ resistant isolate/IC_50_ susceptible isolate) were calculated.

## Results

Adult worms recovered from a hookworm case confirmed as being multiple-drug resistant in this study were identified using morphological criteria as being *A. caninum* (not shown). Additionally, all samples analyzed were assessed with an ITS-2 deep amplicon sequencing assay as described in the methods section, confirming that they were *A. caninum* based upon sequence identity of the generated amplicons. This assay uses a taxonomy-based approach to assess the identity of generated amplicons, based upon a provided reference database. Compared to an example *A. caninum* reference sequence (GenBank: AB751614), generated ITS-2 amplicons had between 96.9–100% sequence identity compared to the reference. Additionally, compared to an example *A. braziliense* reference sequence (GenBank: DQ438050), generated ITS-2 amplicons had 80.6–82.7% sequence identity, further supporting classification as *A. caninum* rather than *A. braziliense*.

### In vitro assays

**Fig. 1:**
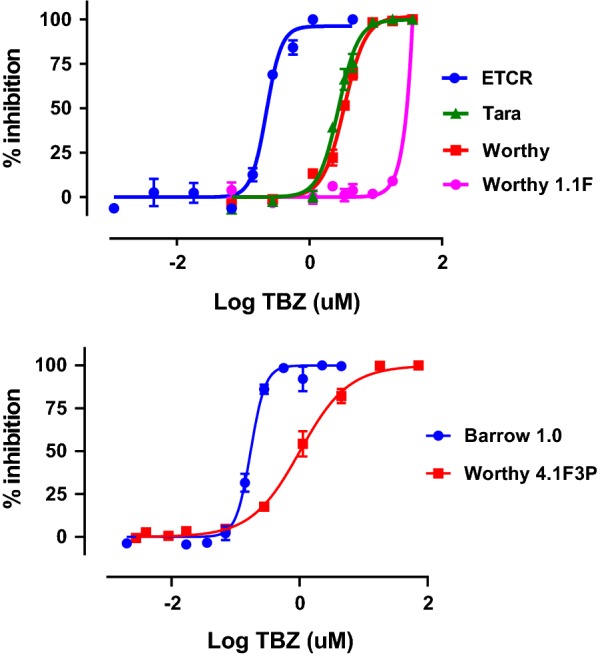
Dose-response curves for the Egg Hatch Assay. Initial assays were performed singly using ETCR, Tara, Worthy and Worthy 1.1F. Subsequent assays were performed in triplicate using the Barrow 1.0 and Worthy 4.1F3P isolates with three replicates per concentration. Curves were generated using the variable slope nonlinear regression model analysis in GraphPad 8

The EHA yielded high *R*^2^ values for the dose response and provided excellent discrimination between the susceptible and resistant isolates. In the initial testing using samples from the original source dogs, the resistance ratios for Lacy, Tara and Worthy, as compared to the ETCR susceptible isolate were 10.9, 11.8 and 14.5, respectively, indicating that these isolates had a high level of resistance to benzimidazole anthelmintics (Fig. 1, Table 2).

**Table 2:** IC_50_ data for benzimidazoles in *Ancylostoma caninum* isolates

Isolate	EHA (µM) IC_50_ (95% CI)	EHA IC_50_ RR	LDA (µM) IC_50_	LDA IC_50_ RR	EHA (µM) IC_95_ (95% CI)	EHA IC_95_ RR
ETCR	0.23		–		0.49	
ETCR 1.0	0.25		0.07		1.13	
Barrow	0.24		–		3.46	
Tara	2.73	11.8	0.12	1.7	7.60	15.5
Lacy	2.51	10.9	0.13	1.8	31.14	63.6
Worthy	3.35	14.5	–		10.07	20.6
Worthy 1.1F	> 36	> 100	–		> 36	> 70
Worthy 2.1F	2.65	11.5	–		> 36	> 70
Barrow 1.0	0.17 (0.16–0.19)	–	–		0.36 (0.28–0.46)	
Worthy 4.1F3P	1.02 (0.92–1.12)	6.0	–		14.85 (9.96–23.22)	41.3

*Notes*: ETCR was the susceptible isolate used for calculating resistance ratios in the initial assays and Barrow 1.0 was used for the EHA and ETCR 1.0 for the LDA in subsequent assays. Initial assays were performed singly using ETCR, ETCR 1.0, Barrow, Tara, Lacy, Worthy, Worthy 1.1F and Worthy 2.1F and subsequent assays were performed in triplicate using Barrow 1.0 and Worthy 4.1F3P, in order to improve the precision of the estimate and reduce the width of the 95% confidence intervals (CI). The values for Barrow 1.0 and Worthy 4.1F3P represent the mean IC_50_ of three biological replicate assays, with each thiabendazole concentration measured in triplicate. IC_95_ values were also calculated for the EHA. Resistance ratios (RR) were calculated as IC_50/95_ resistant isolate/IC_50/95_ susceptible isolate; RR are not provided for the susceptible isolates or where assays were not performed, as this value has no relevance in those instances

*Abbreviations*: EHA, egg hatch assay; LDA, larval development assay; –, assays not performed

Interestingly, a second EHA performed on the first passage of the Worthy isolate 13 days following treatment with fenbendazole demonstrated a large shift in dose response as compared to the original test. The IC_50_ for Worthy increased more than 10-fold, from 3.35 µM to greater than 36 µM, yielding a resistance ratio of greater than 100. An accurate IC_50_ could not be calculated since 36 µM was the highest concentration tested. Subsequent testing using the laboratory isolates Barrow 1.0 and Worthy 4.1F3P, also yielded high *R*^2^ values, but the slope of the dose response for Worthy 4.1F3P had changed as compared to previous assays and this impacted the calculated value for IC_50_. Although the IC_50_ for the susceptible Barrow 1.0 isolate (0.17 µM) was similar to that of the susceptible ETCR isolate, the IC_50_ for Worthy 4.1F3P decreased to 1.01 µM; this yielded a resistance ratio of only 6. In comparison, the resistance ratio for the IC_95_ was 41.25; this difference from the resistance ratio for the IC_50_ is largely due to the difference in the slope of the dose response (Fig. 1, Table 2).

**Fig. 2:**
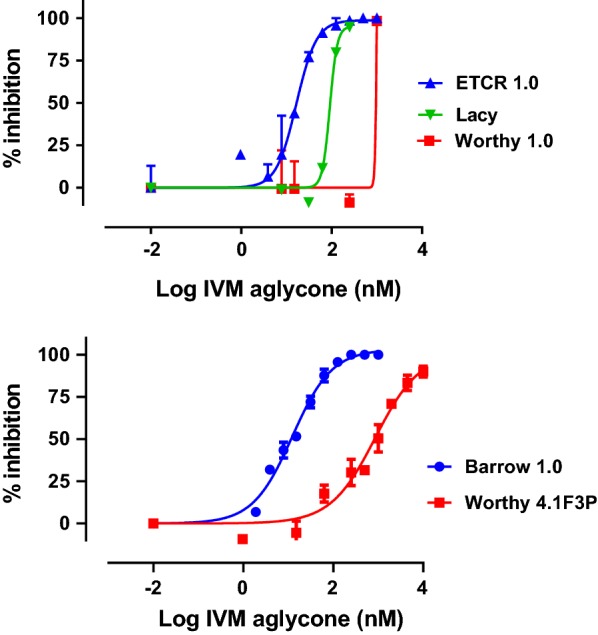
Dose-response curves for the Larval Development Assay. Initial assays were performed singly using ETCR 1.0 Lacy and Worthy 1.0. Subsequent assays were performed in triplicate using Barrow 1.0 and Worthy 4.1F3P isolates with two replicates per concentration. Curves were generated using the variable slope nonlinear regression model analysis in GraphPad 8

The LDA failed to provide good discrimination between the benzimidazole-susceptible and -resistant isolates, yielding resistance ratios of less than 2.0 (Table 2). Using levamisole, the LDA yielded dose response curves with low *R*^2^; this prevented both the calculation of accurate IC_50_ values and any useful discrimination between pyrantel-susceptible and -resistant isolates (data not shown). In contrast, ivermectin aglycone, yielded strong discrimination for detecting resistance to macrocyclic lactones, with resistance ratios of 5.5 and 63.2 for Lacy and Worthy 1.0, respectively (Fig. 2, Table 3).

**Table 3:** DrenchRite LDA dose response data for macrocyclic lactones in *Ancylostoma caninum* isolates

Isolate	LDA IC_50_ (nM) (95% CI)	*R*^2^	LDA RR
ETCR 1.0	16.62	0.93	
Lacy	91.53	0.53	5.5
Worthy 1.0	1052	0.45	63.2
Barrow 1.0	12.31 (10.42–14.70)	0.98	
Worthy 4.1F3P	859 (411.3–3426)	0.92	69.8

*Notes*: Initial assays were performed singly using ETCR 1.0, Lacy and Worthy 1.0 and subsequent assays were performed in triplicate using Barrow 1.0 and Worthy 4.1F3P, in order to improve the precision of the estimate and reduce the width of the 95% confidence intervals (CI). In all assays each ivermectin aglycone concentration was measured in duplicate. Resistance ratios (RR) were calculated as IC_50_ resistant isolate/IC_50_ susceptible isolate; RR are not provided for the susceptible isolates, as this value has no relevance

*Abbreviations*: LDA, larval development assay

Assays performed using multiple biological replicates of Barrow 1.0 and Worthy 4.1F3P yielded high *R*^2^ values for the dose response and a resistance ratio of 69.8, which was quite similar to the resistance ratio for the macrocyclic lactones in the earlier assays (Fig. 2, Table 3).

### In vivo measurements

**Fig. 3:**
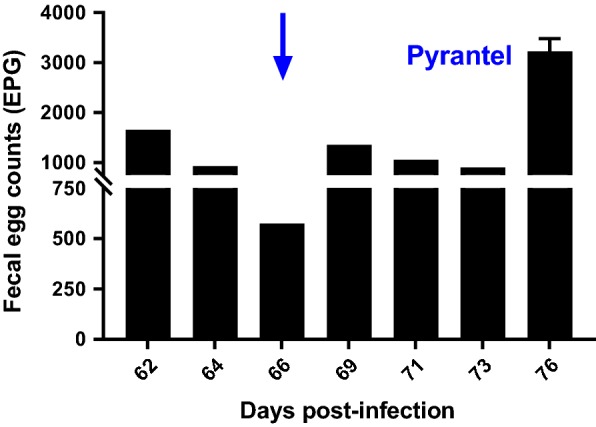
Fecal egg counts (FEC) over the course of infection of a dog infected with the Tara isolate. Treatment with pyrantel was administered on day 66 (23 February 2018) and post-treatment FEC was performed on day 10 post-treatment

**Fig. 4:**
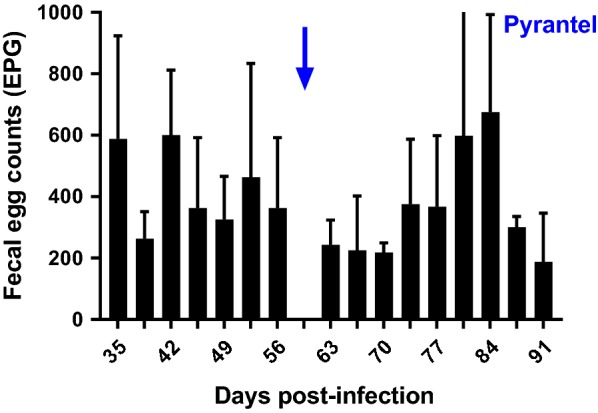
Average of fecal egg counts (FEC) over the course of infection of two dogs infected with larvae from the second passage of the Worthy isolate, with a treatment event with fenbendazole on the first passage (Worthy 2.1F). Treatment with pyrantel was administered on day 55 (25 October 2018) and post-treatment FEC was performed on day 13 post-treatment

Reductions in FEC were measured on the Tara and Worthy isolates following treatment with pyrantel. For both isolates there was essentially no reduction in FEC following treatment with pyrantel; FEC in Tara actually increased (negative reduction) and FEC in Worthy remained unchanged (3% reduction) (Figs. 3, 4, respectively).

### Benzimidazole resistance-associated SNP frequencies determined by deep amplicon sequencing

Two PCR amplicons, encompassing codons 167 and 198/200 of the isotype 1 β-tubulin gene respectively, were sequenced at depth to investigate the presence and determine the frequency of SNP associated with benzimidazole resistance in ruminant trichostrongylid species (Table 4).

**Table 4:** Single nucleotide polymorphism frequencies for *A. caninum* isolates at the three different codons associated with resistance to benzimidazoles

Isolate/Patient	BZ phenotype (S/R)	Sample sequenced	F167Y Freq (%)	E198A Freq (%)	F200Y Freq (%)
ETCR	S	250 eggs	0	0	0
ETCR	S	300 L3	8.8	0	0
Barrow	S	250 L3	1.2	0	0
Worthy	R	L3	92.2	0	0
Worthy 1.1F	R	300L3	87.6	0	0
Worthy 2.1F	R	100 L3	94.5	0	0
Tara	R	375 eggs	12.7	0	0
Tara 1.1F	R	250 L3	50.9	0	0
Lacy	R	Single adult	47.4	0	0
Lacy	R	Single adult	52.9	0	0
Lacy	R	Single adult	46.0	0	0
Lacy	R	Eggs	99.7	0	0
Fame Taker	R	350 L3	90.7	0	0
Dolores (Worthy house companion)	R	300 L3	88.9	0	0

*Abbreviations*: BZ, benzimidazoles; Freq, frequency; L3, third-stage larvae; S/R, susceptible/resistant

SNP associated with benzimidazole resistance were only seen at position 167. All three phenotypically resistant isolates had a high frequency of the benzimidazole resistance associated F167Y (TTC > TAC) SNP in the samples tested, ranging from 13% to almost 100% (Table 4). In the samples from the susceptible isolates, the allele frequencies were 0%, 1% and 9% (Table 4). In the Tara isolate, following a single treatment with fenbendazole the SNP frequency increased from 13% to 51%. For the Lacy isolate, the adults that were expelled after treatment with fenbendazole had allele frequencies of around 50% indicating these worms were heterozygous for the SNP, whereas the eggs recovered from the same feces as the adults had SNP frequencies close to 100%. For the clinical cases Fame taker and Dolores, the SNP frequency was around 90%.

## Discussion

In this study, we conclusively demonstrate for the first time the presence of multiple drug resistance to benzimidazoles, macrocyclic lactones and pyrantel in *A. caninum*. Coincident with our studies, a separate recent study reported resistance to benzimidazoles and macrocyclic lactones in *A. caninum* recovered from a greyhound dog [34]. The origins of these resistant hookworms remain to be determined; however, evidence suggests that they originated from racing greyhound farms. *Ancylostoma caninum* is the most prevalent parasitic nematode in racing greyhounds [50, 51] and this is attributed to the near constant exposure of these dogs to infective third-stage larvae in the sand/dirt exercise run/pens [25]. Racing greyhounds are also treated extremely frequently with multiple different anthelmintics throughout their lives [25]. The intervals between these treatments often are less than the pre-patent period for hookworms. This high intensity of treatment will minimize the amount of refugia (parasite life stages that are not exposed to anthelmintic treatment). Consequently, any worms surviving treatment will have a large reproductive advantage and will rapidly increase in frequency [52]. This combination of factors is known to place heavy selection pressure for drug resistance [24] and is very similar to the epidemiological factors that have led to high levels of multiple-drug resistance in nematodes of sheep and goats, worldwide. The EHA is an *in vitro* bioassay used for detecting resistance to benzimidazole anthelmintics [53]. Based on the ovicidal properties of the benzimidazole drug class [54], this assay has been used successfully to detect resistance against benzimidazoles in multiple nematode parasites of livestock [55–57]. Additionally, the EHA was assessed in *A. caninum* [40] and used to evaluate drug susceptibility/resistance to benzimidazoles in the human hookworm, *Necator americanus* [40, 58, 59]. The IC_50_ values we measured for the two susceptible isolates we tested were very similar to that previously reported for *A. caninum* [40], but in the resistant isolates, there was a clear shift to the right in the dose-response with resistance ratios greater than 6.0 in all isolates tested. Interestingly, when the EHA was repeated on parasite eggs collected from the resistant Worthy 1.0 isolate soon after treatment with fenbendazole, the right shift in the dose response increased dramatically, producing a resistance ratio of greater than 100. Given that the high β-tubulin SNP frequencies measured for Worthy, had no significant change in the SNP frequencies in the before and after treatment samples, this dramatic increase in IC_50_ and resistance ratio suggests that the treatment triggered the induction of another resistance mechanism(s). The fact that the allele frequency did not change and the increase in the levels of resistance was only temporary suggests that was not due to heterogeneity, but instead a change in the parasite population, otherwise, it would have been a permanent change. However, this high level of induced resistance was only temporary, as testing of the same isolate on the second passage produced IC_50_ values similar to the original Worthy isolate prior to fenbendazole treatment. Nevertheless, these observations demand further study. Overall, these data demonstrate clearly that the EHA is able to effectively discriminate between benzimidazole-susceptible and -resistant isolates and that the isolates tested have high levels of benzimidazole resistance.

The LDA is a commonly used *in vitro* bioassay used for detecting resistance to multiple different classes of anthelmintics in gastrointestinal (GI) nematode parasites of sheep and goats [42, 60, 61]. The LDA is based on the ability of anthelmintics to prevent free-living pre-parasitic nematode stages from developing to the infective third-larval stage (L3) [62]. Testing the LDA using multiple isolates of *A. caninum*, both multiple-drug resistant and susceptible, we found the LDA to provide excellent discrimination between our susceptible and resistant isolates for the macrocyclic lactones, but did not provide useful levels of discrimination for benzimidazoles, or for pyrantel. The poor discrimination for resistance to benzimidazoles was similar to that recently reported for *A. caninum* [34]. Thus, unlike for GI nematodes of sheep where the LDA provides good discrimination for multiple drug classes, when used with *A. caninum*, the LDA appears only useful for measuring resistance to macrocyclic lactone drugs. This finding builds on previous works demonstrating that *in vitro* bioassays used for detection of anthelmintic resistance in parasitic nematodes are highly species-specific and drug class-specific in their ability to provide useful levels of discrimination between susceptible and resistant isolates [43, 57, 63].

Interestingly, we found a wide range in the level of resistance in the two resistant isolates we tested and these differences seem to correlate with the clinical case histories of the source dogs prior to our receipt of the samples. The IC_50_ for the first passage of the Worthy isolate yielded a resistance ratio of 63.2, which is more than 11 times greater than the resistance ratio of 5.5 that we measured for Lacy. As noted in the clinical case histories, there was no history of recent use of macrocyclic lactones in Lacy, whereas Worthy had received three consecutive monthly treatments with moxidectin (Advantage Multi®, Bayer, Leverkusen, Germany) just prior to our receipt of the sample. Furthermore, at the time the LDA data were collected, the Worthy isolate had not received treatment with a macrocyclic lactone drug after being established in the laboratory. This difference in clinical history likely is relevant for several reasons. First, to the best of our knowledge, greyhound farms and kennels have been administering ivermectin for parasite control for decades, but did not begin using moxidectin until very recently. Thus, it is unlikely that any of the dogs infected with the resistant isolates evaluated in this study were treated with moxidectin prior to adoption. Secondly, moxidectin is considerably more potent than ivermectin against many nematodes [64]. In *H. contortus*, ivermectin-resistant worms that are naïve to moxidectin are typically killed at very high efficacy following administration of moxidectin [65, 66]; however, once moxidectin is used regularly in an ivermectin-resistant population, resistance to moxidectin can develop rapidly [61]. A study investigating the emergence of moxidectin resistance in *H. contortus* found that a farm naïve to moxidectin but with ivermectin resistance had an LDA resistance ratio of 5.3, whereas farms with resistance to moxidectin had resistance ratios of 32–128, which is 6–24-fold higher [61]. These similarities in the *A. caninum* and *H. contortus* data suggest that the resistant hookworms originating with the greyhounds and now spreading into the pet population have a clinically relevant level of resistance to macrocyclic lactones even without further selection, such as those infecting Lacy. However, as evidenced by the data from Worthy, additional selection with moxidectin can rapidly lead to very high levels of field-derived resistance.

The other recent report of resistance in *A. caninum* [34] also used the LDA to measure resistance to macrocyclic lactones; however, the data of the two studies are dramatically different. The IC_50_ and corresponding resistance ratio we measured in *A. caninum* for both macrocyclic-resistant and -susceptible isolates were fairly comparable to those previously reported for *H. contortus* [61]. However, Kitchen et al. [34] reported values that are vastly different, both in terms of IC_50_ level and in magnitude of resistance ratio. The IC_50_ reported for their resistant isolate was lower than what we measured in our susceptible isolate and the IC_50_ reported for their susceptible isolate was at pM levels, almost 5000-fold lower than what we measured. This yielded resistance ratio of greater than 1000; a level that is greater than what has been reported, even in the most resistant *Haemonchus* isolates. Given the available clinical histories, the resistant isolate they studied was likely similar to the Lacy isolate, with little to no previous exposure to moxidectin. We measured a 5.5 resistance ratio for the Lacy isolate, thus their analyses demonstrated a resistance ratio more than 200 times greater than what we measured. Additionally, we consistently generated sigmoidal dose response curves with high *R*^2^ and readily achieved 100% inhibition of development for our susceptible isolate. In contrast, data shown in Kitchen et al. [34] indicate that inhibition greater than 80% was not achieved and shapes of dose response curves were not sigmoidal. The cause of these differences is not readily apparent, but these are likely due to differences in the methods used in the two studies.

An additional interesting observation was that following treatment with fenbendazole, the egg counts in dogs infected with both the Tara and Worthy isolates initially decreased by greater than 99%, but then steadily increased after treatment to rather high levels (Additional file 1: Table S1). Additionally, the mild clinical signs of enteritis that one of the dogs was displaying prior to treatment did not improve post-treatment. Given the EHA and β-tubulin SNP frequency data demonstrating extremely high levels of resistance in the surviving worms, the egg count and clinical response data suggest that the treatment was poorly effective in killing the worms, but induced a temporary inhibition of egg production. A similar temporary deleterious effect on worm fecundity has been reported previously for benzimidazoles in *H. contortus* in sheep [67], but is not recognized as an usual effect in nematodes of livestock following treatment with benzimidazoles. In contrast, this phenomenon has been reported on multiple occasions following treatment with ivermectin and moxidectin [68–70]. Regarding the reductions in FEC measured for pyrantel, for both isolates, it was clear that there was no effect of treatment (Figs. 3, 4).

Currently, the mechanisms of resistance to macrocyclic lactones and pyrantel in nematodes are unknown. Consequently, there are no molecular diagnostics available to detect resistance to these drug classes. However, the mechanism of resistance to benzimidazole drugs is well described. Benzimidazoles work by blocking the polymerization of parasite microtubules and they do this by binding to the nematode β-tubulin protein monomers [71, 72]. SNPs in the isotype-1 β-tubulin gene located at codons 167 (TTC/Phe→TAC/Tyr), 198 (GAG/Glu→GCG/Ala) and 200 (TTC/Phe→TAC/Tyr) are associated with benzimidazole resistance in multiple species of strongylid nematode parasites such as *Haemonchus contortus* [72], *Teladorsagia circumcincta* [73] and cyathostomins [74]. Several PCR and pyrosequencing assays have been developed to detect and measure these mutations, [75–81] but these all have limitations that affect their usefulness.

However, a recently developed deep-amplicon sequencing assay for measuring benzimidazole-associated resistance mutations in nematode communities of cattle, sheep, bison and horses provides a powerful new tool that enables unparalleled sensitivity of detection and permits screening for the emergence of resistance mutations [48]. We modified and used this deep amplicon-sequencing assay in *A. caninum* and here we report, to the best of our knowledge, the first use of this approach in a hookworm. Of the SNP associated with benzimidazole resistance in trichostrongylid nematodes, only F167Y (TTC > TAC) was detected. This same SNP has been commonly found in other strongylid nematode parasites such as equine cyathostomins [82], *Haemonchus contortus* [83], *H*. *placei* [84] and *Teladorsagia circumcincta* [85], and has only been rarely reported in *Ascaris lumbricoides* and *Trichuris trichuira* [40]. Recently, this same SNP was also reported in a resistant isolate of *A. caninum* that was originally isolated from a racing greyhound from Florida. Furthermore, using CRISPR/Cas 9, Kitchen et al. [34] were successful in replicating this SNP in the homologous *ben-1* gene of *C*. *elegans* and saw a doubling of the resistance ratio in the EHA, which was similar to the resistance ratio measured in their *A. caninum* resistant isolate using the LDA [34].

Using deep amplicon sequencing, we found low allele frequencies for the benzimidazole resistance-associated SNPs in the susceptible isolates; in Barrow, the frequency was 1.2% and the two analyses for ETCR yielded highly variable results of 0 and 8.8%. The reason for this discrepancy is not known and further analyses are in progress. In contrast, high SNP frequencies were recorded for all resistant isolates. The original isolate of Worthy had a SNP frequency of 92.2%, which is consistent with the high selection pressure produced by the five rounds of intensive (10-day) fenbendazole treatment the dog received in the year prior to us collecting the sample. The lowest frequency measured in a resistant isolate was 12.7% in Tara, however, following a single treatment with fenbendazole, the SNP frequency increased to 50.9%. It is unclear why Tara had a relatively low SNP frequency relative to the other resistant isolates, given that Tara had a history of multiple treatments with febantel prior to our receipt of the sample. Further analyses are in progress with all of our archived samples to address these issues. Interestingly, three single adult worms recovered from the feces of Lacy that we sequenced (out of many that were expelled alive after treatment with fenbendazole) had F167Y (TTC > TAC) SNP frequencies of approximately 50% indicating that these worms were heterozygous at codon 167. This was an interesting finding, as it suggests that heterozygous worms were able to survive the treatment, but could not maintain their position in the gastro-intestinal tract. In comparison, eggs recovered from the same feces demonstrated a SNP frequency of almost 100%, suggesting that the worms that survived the treatment and maintained their position in the intestine were virtually all homozygous for resistance.

It is noteworthy that others have looked for benzimidazole resistance-associated SNP in *A. caninum* without success [86]. However, studies performed in Brazil did report finding a SNP at codon 198 in *A. braziliense* [87] and at codon 200 in *A. caninum* [88] at very low frequencies, 1.2 and 0.8%, respectively, using PCR-RFLP. However, these findings were not confirmed by sequencing.

Here we report compelling evidence using *in vitro*, *in vivo* and genetic analyses that convincingly demonstrate that recent cases of hookworm in dogs that appear refractory to treatment, are due to *A. caninum* that are MDR. Although larval leak is likely involved in most of these cases, our data indicate strongly that MDR is the primary cause. This is an important and concerning development, as the emergence and spread of MDR *A. caninum* to all three major anthelmintic classes, would pose a serious threat to canine health, as there are no other effective drug classes currently approved for the treatment of hookworms in dogs in the USA. Although a recent study reported success in treating several cases of recurrent hookworm infections in greyhounds recently retired from racetracks using a combination therapy of moxidectin, pyrantel pamoate and febantel at monthly intervals [89], we have recently diagnosed multiple cases at a greyhound adoption kennel where this same regimen appears to be ineffective (data not shown). The disparity in these findings is consistent with the rapid evolution of moxidectin resistance when moxidectin is used against ivermectin resistant worms [61].

## Conclusions

MDR in *A. caninum* is an emerging problem in dogs. Evidence suggests that the problem originated in the greyhound racing industry and has since begun to spread through the pet population. Nevertheless, we still lack definitive evidence to infer that these resistant hookworms are spreading into the pet dog population. Clearly, further epidemiological and molecular epidemiological investigations are needed in order to gain knowledge on the origin, prevalence and distribution of MDR *A. caninum*. Furthermore, new treatments approved for use in dogs are greatly needed. These results also provide proof of concept that anthelmintic resistance can arise in hookworm species. *Ancylostoma caninum* is extremely close phylogenetically to the hookworm species of humans, *A*. *duodenale*, *A*. *ceylanicum* and *Necator americanus* [90]. Consequently, these findings should provide some concern to the global health community, as the scale-up of mass drug administration for soil-transmitted helminths (STH) is now placing similar selection pressures for benzimidazole resistance in human hookworms and reduced efficacies are widely reported [91–96]. The deep amplicon sequencing assay used in this study, also can be used to perform global-level surveillance for the detection of benzimidazole resistance in human hookworms and with minor modifications, in roundworms (*Ascaris lumbricoides*) and whipworms (*Trichuris trichiura*).

## Supplementary information

**Additional file 1: Table S1.** Fecal egg count reduction (FECR) data for fenbendazole, different post-treatment timepoints were tested. Fecal egg count reduction was calculated using the following formula: (Pre-treatment FEC – Post-treatment FEC) / (Pre-treatment FEC) × 100. Experimental infections in one dog each for the Tara and Worthy isolate.

## Data Availability

The datasets supporting the conclusion of this article are included within the article and its additional file. The raw molecular data can be accessed *via* the following DOI link https://www.ncbi.nlm.nih.gov/sra/PRJNA556272.

## Abbreviations

AR: antihelminthic resistance
EHA: egg hatch assays
LDA: larval developmental assays
FEC: fecal egg counts
FECR: fecal egg count reduction
ITS-2: internal transcribed spacer 2
MDR: multiple-drug resistant
SNP: single nucleotide polymorphisms
