# Supplementary material for: Multiple drug resistance in the canine hookworm Ancylostoma caninum: an emerging threat?
Source: Parasit Vectors. 2019 Dec 9;12:576. doi: 10.1186/s13071-019-3828-6 (PMC6902405; doi:10.1186/s13071-019-3828-6)
Supplement: Supplementary file 1 — Additional file 1: Table S1. Fecal egg count reduction (FECR) data for fenbendazole, different post-treatment timepoints were tested. Fecal egg count reduction was calculated using the following formula: (Pre-treatment FEC – Post-treatment FEC) / (Pre-treatment FEC) × 100. Experimental infections in one dog each for the Tara and Worthy isolate. [file 13071_2019_3828_MOESM1_ESM.docx]

**Additional file 1. Table S1.** Fecal egg count reduction (FECR) data for fenbendazole, different post-treatment timepoints were tested. Fecal egg count reduction was calculated using the following formula: ((Pre-treatment FEC – Post-treatment FEC) / (Pre-treatment FEC)) x 100. Experimental infections in one dog each for the Tara and Worthy isolate.

| Day Post-Tx | % FECR | |
| --- | --- | --- |
|  | **Tara** | **Worthy** |
| 3 | **99.7** | **99.7** |
| 13 | **91.7** | **90.2** |
| 23 | **64.0** | **85.9** |
